# Cost-effectiveness of malaria preventive treatment for HIV-infected pregnant women in sub-Saharan Africa

**DOI:** 10.1186/s12936-017-2047-x

**Published:** 2017-10-06

**Authors:** Sung Eun Choi, Margaret L. Brandeau, Eran Bendavid

**Affiliations:** 1 Department of Management Science and Engineering, Stanford University, Stanford, CA USA; 2 Division of General Medical Disciplines, Stanford University, Stanford, CA USA; 3 Center for Health Policy and the Center for Primary Care and Outcomes Research, Stanford University, Stanford, CA USA

## Abstract

**Background:**

Malaria is a leading cause of morbidity and mortality among HIV-infected pregnant women in sub-Saharan Africa: at least 1 million pregnancies among HIV-infected women are complicated by co-infection with malaria annually, leading to increased risk of premature delivery, severe anaemia, delivery of low birth weight infants, and maternal death. Current guidelines recommend either daily cotrimoxazole (CTX) or intermittent preventive treatment with sulfadoxine–pyrimethamine (IPTp-SP) for HIV-infected pregnant women to prevent malaria and its complications. The cost-effectiveness of CTX compared to IPTp-SP among HIV-infected pregnant women was assessed.

**Methods:**

A microsimulation model of malaria and HIV among pregnant women in five malaria-endemic countries in sub-Saharan Africa was constructed. Four strategies were compared: (1) 2-dose IPTp-SP at current IPTp-SP coverage of the country (“2-IPT Low”); (2) 3-dose IPTp-SP at current coverage (“3-IPT Low”); (3) 3-dose IPTp-SP at the same coverage as antiretroviral therapy (ART) in the country (“3-IPT High”); and (4) daily CTX at ART coverage. Outcomes measured include maternal malaria, anaemia, low birth weight (LBW), and disability-adjusted life years (DALYs). Sensitivity analyses assessed the effect of adherence to CTX.

**Results:**

Compared with the 2-IPT Low Strategy, women receiving CTX had 22.5% fewer LBW infants (95% CI 22.3–22.7), 13.5% fewer anaemia cases (95% CI 13.4–13.5), and 13.6% fewer maternal malaria cases (95% CI 13.6–13.7). In all simulated countries, CTX was the preferred strategy, with incremental cost-effectiveness ratios ranging from cost-saving to $3.9 per DALY averted from a societal perspective. CTX was less effective than the 3-IPT High Strategy when more than 18% of women stopped taking CTX during the pregnancy.

**Conclusion:**

In malarious regions of sub-Saharan Africa, daily CTX for HIV-infected pregnant women regardless of CD4 cell count is cost-effective compared with 3-dose IPTp-SP as long as more than 82% of women adhere to daily dosing.

**Electronic supplementary material:**

The online version of this article (doi:10.1186/s12936-017-2047-x) contains supplementary material, which is available to authorized users.

## Background

HIV and malaria are leading causes of disease burden in sub-Saharan Africa; combined, the two diseases cause nearly 20% of all deaths annually [1, 2]. While the geographical epicenters of the two diseases are different—malaria is most common in Central and West Africa, where HIV prevalence is relatively low, and HIV is most prevalent in Southern Africa, where the malaria burden is lower—the overlap is substantial, especially in Eastern Africa, as is the burden of co-infection [3]. Each disease potentially exacerbates the other: HIV-infected adults are at increased risk of complicated and severe malaria and death, while malaria results in increased viral load and may speed disease progression and spread [4].

Malaria is an important cause of morbidity and mortality among HIV-infected pregnant women in co-endemic regions of sub-Saharan Africa, where at least 1 million malaria co-infections occur among pregnant women annually [5]. HIV-infected pregnant women who experience clinical malaria are at increased risk of premature delivery, severe anaemia, delivery of low birth weight infants, and death [6, 7].

Guidelines currently encourage providing pregnant women with malaria preventive treatment during pregnancy in malaria-endemic countries [8, 9]. Previous guidelines recommended 3 doses of intermittent preventive treatment with sulfadoxine–pyrimethamine (IPTp-SP) for HIV-infected pregnant women with CD4 > 350 cells/mm^3^ or daily cotrimoxazole (CTX) for women with CD4 < 350 cells/mm^3^ [10]. Despite these guidelines, median coverage rate of receiving at least 1 dose of IPTp-SP from antenatal clinics in sub-Saharan Africa is 19.0%, which remains markedly lower than the international targets, despite high rates of antenatal clinic attendance (75% of women in sub-Saharan Africa attend at least twice) [11, 12]. In analyses of the low coverage of IPTp-SP in sub-Saharan Africa, information barriers (unclear guidelines and uncertain messages about timing of IPTp-SP in pregnancy), behavioral barriers (irregular antenatal clinic visits), and system barriers [sulfadoxine–pyrimethamine (SP) stockouts and low clinic staffing] emerge as salient [13–18].

In 2014, the World Health Organization (WHO) provided updated guidelines that recommend CTX for pregnant women with severe or advanced HIV clinical disease (WHO stages 3 or 4) or CD4 cell counts below 350 cells/mm^3^ and, in contexts where malaria or severe bacterial infections are common, CTX regardless of CD4 cell count or HIV severity [8].

Implementation of the WHO guidelines remains mixed. A cross-sectional analysis of observational data in Malawi found that daily CTX was associated with reduced malaria parasitaemia and anaemia compared to IPTp-SP among HIV-positive pregnant women [19]. However, while CTX was found to be effective in preventing clinical malaria [20, 21], long-term efficacy relative to standard IPTp-SP, toxicity, and birth outcomes associated with CTX have not been established in trials [22]. Many national guidelines still recommend a mix of IPTp-SP and CTX because of past WHO guidelines that recommended IPTp-SP, and because imperfect adherence to daily CTX may put women at risk compared with only 3 doses of IPTp-SP [23].

A model-based analysis to assess the effectiveness and cost-effectiveness of daily CTX relative to IPTp-SP among HIV-infected pregnant women in sub-Saharan Africa was performed.

## Methods

An individual-level microsimulation model of malaria among HIV-infected pregnant women was constructed and parameterized to five sub-Saharan countries: Ghana, Kenya, Malawi, Mozambique, and Tanzania. These countries were selected because they are all co-endemic, with wide ranges of HIV and malaria burdens.

Cohorts of 10,000 HIV-infected pregnant women from each country were simulated over a 40-week pregnancy period. Each woman was characterized by CD4 cell count, antiretroviral therapy (ART) status, and malaria preventive treatment status. Tables 1 and 2 summarizes key model parameters and data sources, further detailed in the Additional file 1, consistent with international model reporting guidelines [24]. Here, an overview of the model’s key components is provided.

**Table 1:** Model parameters

Variable	Value	Distribution	Source
Demographic			
Malaria prevalence in HIV-infected pregnant women	31.0%	Beta(3.1,6.9)	[46]
CD4 count	390	Normal(390,220)	[47]
Incidence			
Relative risk of malaria in HIV-infected pregnant woman	1.6	Gamma(1.6,1)	[48]
CD4 change while on ART	70	Normal(70,100)	[49]
Mortality			
Neonatal mortality risk due to low birth weight	6.93%	Beta(0.007,0.093)	[30]
Annual mortality due to malaria during pregnancy	0.33%	Beta(0.033,9.967)	[30]
Annual mortality due to anaemia in pregnancy	1.0%	Beta(0.1,9.9)	[30]
Annual HIV mortality among HIV-infected pregnant women	0.0161%	Beta(0.16,9.84)	[50]
Annual HIV mortality among HIV-infected pregnant women on ART	0.007%	Beta(0.07,9.93)	[47, 50]
Relative risk of HIV mortality of CTX while on ART	0.47	Beta(8,9)	[35]
DALY calculations			
Annual discount rate	3%	3%	[51]
Average age (years)	22.83	22.83	[29]
Life expectancy for women aged 20–24 (years)	47.24	Normal(47,3)	[32]
Life expectancy at birth (years)	57.96	Normal(58,3)	[32]
Disability weight—malaria during pregnancy	0.21	Normal(0.21,0.03)	[31]
Disability weight—maternal anaemia due to malaria	0.06	Normal(0.06,0.02)	[31]
Disability weight—low birth weight	0.11	0.11	[33]
Length of disability (years)—malaria during pregnancy	0.01	Gamma(2.0,0.004)	[30]
Length of disability (years)—maternal anaemia due to malaria	0.06	Gamma(1.2,0.04)	[52]
Length of disability (years)—low birth weight	57.96	Gamma(32,1.8)	[32]
Costs (USD 2015)			
Drug cost			
Intermittent preventive treatment (per dose)	$0.20	Normal(0.2,0.01)	[30, 53]
Cotrimoxazole, 480 mg twice daily (per year)	$6.28	Gamma(3.9,1.61)	[54]
Healthcare labor cost^a^			
Labor time per IPTp-SP administration (min)	8.31	Gamma(13.85,0.6)	[30]
Nurses’ monthly cost of labor	542.76	Gamma(29.3,18.5)	[30]
Household cost			
Antenatal care visit direct cost	0.47	Gamma(92.4,0.005)	[30]
Antenatal care visit indirect cost	1.17	Gamma(146,0.008)	[30]
ART treatment (per year)	$193.61	Gamma(2,97)	[55]
Efficacy			
Low birth weight			
Relative risk baseline (IPTp-SP 0–1 dose) vs. 2 dose IPTp-SP	3.25	Gamma(1.1,2.95)	[45]
Incidence per 1000 women given IPTp-SP 2 doses^b^	175	(91–222)^a^	[29]
Relative risk IPTp-SP 3 doses vs. 2 doses	0.86	Normal(0.86,0.21)	[29]
Relative risk CTX vs. 2 dose IPTp-SP	1.16	Gamma (1.05,1.1)	[34, 56]
Maternal parasitaemia			
Relative risk baseline (IPTp-SP 0–1 dose) vs. 2 dose IPTp-SP	1.4	Gamma(1.15,1.2)	[19]
Incidence per 1000 women given IPTp-SP 2 doses^b^	112	(0–359)^a^	[29]
Relative risk IPTp-SP 3 doses vs. 2 doses	0.26	Normal(0.26,0.08)	[29]
Relative risk CTX vs. 2 dose IPTp-SP	0.44	Lognormal(−1.05,0.7)	[19, 34]
Relative risk CTX + ART vs. CTX	0.38	Lognormal(−1.2,0.7)	[21]
Anaemia			
Relative risk baseline (IPTp-SP 0–1 dose) vs. 2 dose IPTp-SP	1.03	Gamma(0.85,1.2)	[45]
Incidence per 1000 women given IPTp-SP 2 doses^b^	582	(333–795)^a^	[29]
Relative risk IPTp-SP 3 doses vs. 2 doses	582	Normal(0.96,0.07)	[29]
Relative risk CTX vs. 2 dose IPTp-SP	0.72	Normal(0.72,0.05)	[19]

*ART* antiretroviral therapy for HIV, *CTX* cotrimoxazole, *DALY* disability-adjusted life year, *IPTp-SP* intermittent preventive treatment with sulfadoxine–pyrimethamine

^a^Labor cost calculations assume that maximum hours of work is 48 h per week, 4.3 weeks per month [57]

^b^Age-adjusted incidence per 1000 women (the range is provided to illustrate low and high risk) under the 2-IPT Low Strategy (the Reference Strategy)

**Table 2:** Model parameters varied across countries

Country	Annual incidence of malaria (per 1000 women) [25]	% of women receiving IPTp-SP [11]	% of pregnant women living with HIV who received ART [58]
Ghana	127	58.2	62
Kenya	63	35.5	63
Malawi	174	80.7	79
Mozambique	262	31.4	84
Tanzania	32	58.4	73

*ART* antiretroviral therapy for HIV, *IPTp-SP* intermittent preventive treatment with sulfadoxine–pyrimethamine

### Treatment strategies

The Reference Strategy modeled receipt of 2 doses of IPTp-SP when CD4 is above 350 cells/mm^3^, and daily CTX when CD4 is below 350 cells/mm^3^. The portion of the model population (HIV-infected pregnant women) receiving these preventive options in each country reflected the current IPTp-SP coverage rate in that country and, therefore, represented common practice in many at-risk populations [25]. Three alternative strategies for reducing risk of malaria in pregnant women were simulated in addition to the Reference Strategy: (1) 3 doses of IPTp-SP if CD4 cell count > 350 and daily CTX if CD4 cell count < 350, at the current IPTp-SP coverage in the country of interest (3-IPT Low Strategy) [26]; (2) same as the 3-IPT Low Strategy, but at a higher coverage equivalent to current ART coverage rates (3-IPT High Strategy); and (3) daily CTX regardless of CD4 cell count (CTX Strategy).

The 3-IPT Low Strategy reflects the possibility that the current capacity to provide IPTp-SP remains unchanged, but adherence to the policy of providing 3 doses (rather than 1 or 2) is improved. In the 3-IPT High Strategy, we assumed that IPTp-SP and CTX coverage rates would reach the current ART coverage rate of the country. ART coverage rates were used as the target coverage rates for IPTp-SP in this strategy, assuming that malaria prevention services could be provided through the same channels that provide HIV care for pregnant women. In the CTX Strategy, the CTX coverage rate was assumed to be the same as the ART coverage rate: women receiving antenatal HIV care were assumed to receive CTX at the same time, regardless of CD4 cell count. It was assumed that in the CTX Strategy, pregnant women with CD4 cell count < 350 would be on CTX at the beginning of their pregnancy (assuming they were placed on CTX prior to pregnancy), and those with CD4 cell counts > 350 would receive CTX starting in the second trimester. In the model, pregnant women with CD4 cell counts > 350 started IPTp-SP in the second trimester and thus received no malaria prophylaxis in the first trimester [27].

In all simulated strategies, it was assumed that all women were eligible to receive Option B+, the current recommended strategy for prevention of mother-to-child HIV transmission [28]. Under Option B+, all HIV-infected pregnant women receive ART regardless of their CD4 cell counts.

### Treatment effectiveness

To estimate the effectiveness of 2 versus 3 IPTp-SP doses, published meta-analytic estimates were used for the reduction in the incidence of primary health outcomes, based on synthesized data from seven trials in six countries [29]. The outcomes included in our analysis were maternal anaemia (haemoglobin < 60 g/L), maternal malaria parasitaemia (blood films found to be positive for *Plasmodium* parasites), and low birth weight (LBW < 2500 g). The morbidity and mortality associated with each outcome were used to estimate the disability-adjusted life year (DALY) implications of each complication based on estimates from a systematic analysis done for the Global Burden of Disease Study [30–33]. For CTX compared to IPTp-SP, treatment effect estimates of the same outcomes were used based on a meta-analysis and a study of HIV-infected pregnant women in Malawi [19, 34].

Compared to 2 doses of IPTp-SP, the relative risk estimates of LBW, anaemia, and malaria parasitaemia for 3 doses of IPTp-SP were 0.86 (95% CI 0.53–1.39), 0.96 (95% CI 0.87–1.07), and 0.26 (95% CI 0.15–0.46), and for CTX were 1.1 (95% CI 0.86–1.34), 0.72 (95% CI 0.61–0.83), and 0.43 (95% CI 0.19–1.10), respectively (Tables 1, 2) [29]. In addition, HIV-infected women receiving both CTX and ART had lower mortality compared to women on ART without CTX when their CD4 cell counts were < 350 [35]; this mortality benefit was varied in sensitivity analysis.

### Costs and utilities

The treatment (drug) cost, healthcare labor cost, and household costs were included. Healthcare labor costs included the value of nurses’ time to administer IPTp-SP and/or CTX assuming estimated labour time per IPTp-SP administration and nurse’s monthly cost of labour. Household costs included direct costs of antenatal care clinic visits and indirect costs of transportation and time spent travelling to and waiting at the health facility. Due to lack of available data on costs associated specific health outcomes modeled in this study, cost savings from averting the malaria complications were not included. All costs were converted to 2015 US dollars using the Consumer Price Index [36].

Health effects were tallied in terms of DALYs averted for the primary health outcomes (LBW, anaemia, and malaria parasitaemia) between competing treatment strategies. The primary outcome of the analysis was the cost per DALY averted. Disutility of disease states to calculate DALYs were based on estimates from the Global Burden of Disease Study and WHO [31–33]. Incremental cost per DALY averted were calculated between competing treatment strategies. All costs and DALYs were discounted at 3% annual rates [37]. A willingness to pay (WTP) of less than each country’s gross domestic product (GDP) per capita was used as a threshold to determine cost-effectiveness [38].

### Sensitivity analyses

One-way sensitivity analyses were performed to investigate the effects of changes in model parameters on estimated outcomes across a wide range of values, including risk of the three primary health outcomes, CTX effectiveness, and adherence to daily CTX. In the base case scenario, CTX was significantly more effective than IPTp-SP in reducing the risk of anaemia and malaria parasitaemia; two-way sensitivity analysis was conducted on the risk of anaemia and malaria parasitaemia to assess sensitivity of results to changes in CTX effectiveness.

In the base case scenario, pregnant women experienced lower mortality from receiving CTX and ART concurrently than receiving ART only when their CD4 cell counts were < 350 cells/mm^3^. These mortality benefits were excluded in sensitivity analysis to isolate the benefits associated with the three primary health outcomes.

IPTp-SP is recommended starting as early as possible in the second trimester while CTX can be administered during the first trimester. In sensitivity analysis, the impact of initiating CTX therapy in the second trimester and how the benefits from CTX would be expected to change relative to the 3-IPT High Strategy when the CTX benefits start to accrue at the same time as IPTp-SP were examined.

Additionally, a probabilistic sensitivity analysis was conducted. For each scenario, the model was re-run 10,000 times while repeatedly Monte Carlo sampling from the probability distributions of all input parameters (Tables 1, 2), generating 95% confidence intervals around all outcomes as per ISPOR guidelines [39]. All analyses were performed in *R* (version 3.2.1, The R Foundation for Statistical Computing, Vienna) using the computer code linked in the Additional file 1 [40–43].

### Role of the funding source

The funders of the study had no role in study design, data collection, data analysis, data interpretation, or writing of the report. The corresponding author had full access to all the data in the study and had final responsibility for the decision to submit for publication.

## Results

### Base case results

**Fig. 1:**
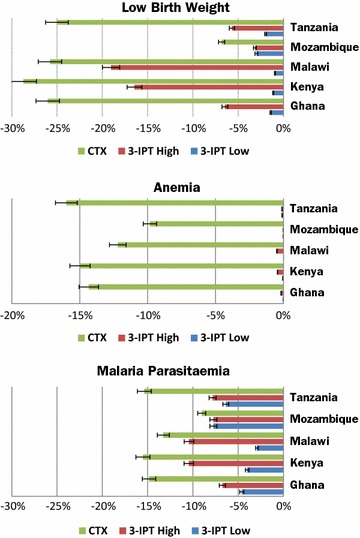
Projected reduction in primary health outcomes, %. The horizontal bars represent projected percentage reduction in incidence of each primary health outcome for different strategies compared to the Reference Strategy (2-dose IPTp-SP)

Figure 1 shows projected reductions in the primary outcomes (LBW, anaemia, and malaria parasitaemia) for each strategy relative to the Reference Strategy in each of the studied countries. Both the CTX and 3-IPT High Strategies substantially reduced the incidence of the three health outcomes in all countries compared to the Reference Strategy. The largest reductions accrued from the CTX Strategy (partly due to the preventive benefits in the first trimester, tested in sensitivity analysis below), and relatively higher coverage rate of CTX compared to IPT in low coverage strategies). Compared to the Reference Strategy, the CTX Strategy reduced incidence of LBW by 6.8% (95% CI 4.2–9.4%) to 28.7% (95% CI 21.7–35.7), anaemia by 9.8% (95% CI 7.2–12.4) to 14.5% (95% CI 8.0–21.9), and malaria parasitaemia by 9.0% (95% CI 6.4–11.6) to 15.5% (95% CI 8.5–22.5) across the five countries.

Base case cost-effectiveness results are presented in Table 3. In all countries, the 3-IPT High Strategy was strictly dominated by the CTX Strategy, which averted more DALYs at lower cost. The 3-IPT Low Strategy was eliminated from consideration by extended dominance; the incremental cost-effectiveness ratio (ICER) of the 3-IPT Low Strategy was higher than that of the CTX Strategy compared to the Reference Strategy except in Mozambique. Compared to the Reference Strategy, the CTX Strategy resulted in ICERs ranging from $0.4 to $3.9 per DALY averted in countries other than Mozambique. In Mozambique, due to relatively higher baseline IPT coverage and higher risk of malaria compared to other studied countries, the 3-IPT Low Strategy was strictly dominated by the CTX Strategy, and as compared to the Reference Strategy, the CTX was cost saving (averting more DALYs at lower cost). DALYs averted from the CTX Strategy compared to any of the IPTp-SP strategies primarily accrued from pregnant women receiving benefits from CTX earlier than IPTp-SP since preventive benefits started during the first trimester for CTX and during the second trimester for IPTp-SP.

**Table 3:** Base case results

Country and strategy	Costs per 10,000 women (USD)	DALYs per 10,000 women	Incremental cost	Incremental DALYs averted	ICER
Ghana					
2-IPT Low^a^	359,992	12,055			
3-IPT Low	360,340	11,912	348	143	Dominated^b^
3-IPT High	361,594	11,434	1602	621	Dominated
CTX	361,009	9312	1017	2743	$0.37
Malawi					
2-IPT Low^a^	393,772	14,671			
3-IPT Low	394,744	14,563	972	108	Dominated^b^
3-IPT High	399,172	12,382	5400	2289	Dominated
CTX	396,580	11,317	2808	3354	$0.84
Kenya					
2-IPT Low^a^	383,217	7000			
3-IPT Low	384,314	6934	1097	66	Dominated^b^
3-IPT High	388,568	6183	5351	817	Dominated
CTX	386,518	5339	3301	1661	$1.99
Mozambique					
2-IPT Low^a^	406,628	16,424			
3-IPT Low	409,130	16,022	2502	402	Dominated
3-IPT High	409,367	15,997	2739	427	Dominated
CTX	404,816	15,240	−1812	1184	Cost-saving
Tanzania					
2-IPT Low^a^	367,892	4364			
3-IPT Low	370,519	4327	2627	37	Dominated^b^
3-IPT High	371,468	4231	3576	133	Dominated
CTX	371,154	3516	3262	848	$3.85

*CTX* cotrimoxazole, *DALY* disability-adjusted life year, *ICER* incremental cost-effectiveness ratio (cost per DALY averted) compared to the strategy in the row above, *IPT* intermittent preventive treatment with sulfadoxine–pyrimethamine

^a^Reference Strategy

^b^Dominated by extended dominance: a linear combination of two other strategies yields greater benefit at equal cost

### Results of sensitivity analyses

**Fig. 2:**
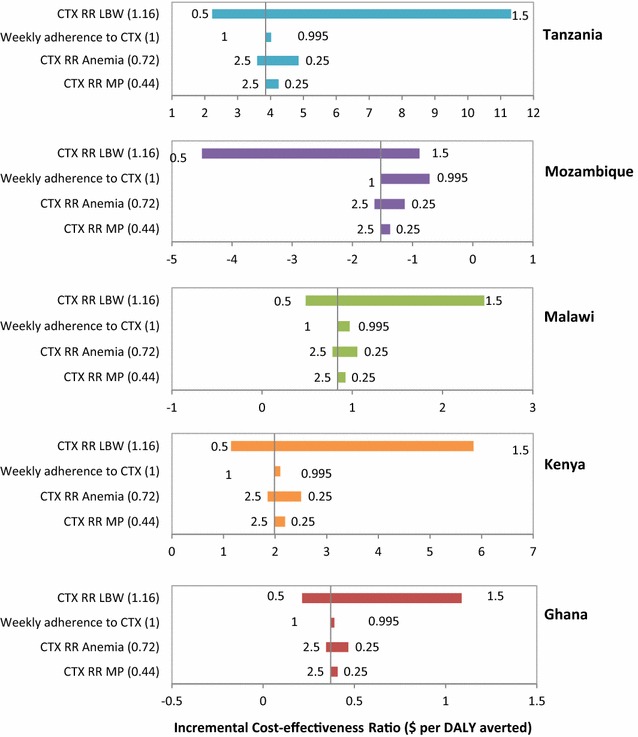
One-way sensitivity analyses: CTX Strategy compared to Reference Strategy. The vertical lines represents the ICER in the base case analysis (2015 US dollars per DALY averted) and the horizontal bars represent the variation of the ICER given variations of key parameters. The numbers at each end of the bars represent the lower and upper bounds of the value used for each parameter. Numbers in brackets represent the deterministic value of each parameter (CTX RR estimates are compared to 2-dose IPTp-SP). *CTX* cotrimoxazole, *RR* relative risk, *LBW* low birth weight, *MP* malaria parasitaemia

Results of sensitivity analyses on CTX effectiveness in reducing the incidence of the three primary health outcomes are summarized in Fig. 2, which shows ICERs of the CTX Strategy compared to the Reference Strategy.

The effects of results of changes in CTX effectiveness in reducing the incidence of the three primary health outcomes would affect results were assessed. Results were most sensitive to changes in CTX effectiveness in reducing LBW incidence. Compared to the Reference Strategy, when the relative risk estimate of CTX relative to 3-IPT for LBW increases by 10% or more (i.e., benefits of CTX decline), the CTX Strategy no longer dominates the 3-IPT High Strategy. Results were not substantially affected by varying the effectiveness of CTX in reducing the incidence of maternal anaemia and malaria parasitaemia over the range of plausible values.

Effects of adherence to daily CTX on health outcomes and cost-effectiveness were evaluated. Perfect adherence to CTX was assumed in the base case. In high-incidence countries (Malawi and Mozambique), when more than 0.5% of pregnant women did not adhere to CTX every week (thus, more than 18% of women dropped out over the pregnancy period), the 3-IPT High Strategy became the preferred strategy, averting more DALYs than the CTX Strategy with ICERs ranging from $2.6 to $13.5 at varying weekly CTX dropout rates (Additional file 1: Table S1 and Figure S2). In lower-incidence countries, when more than 1.5% (Kenya) to 2.5% (Ghana and Tanzania) of pregnant women dropped out weekly (thus, more than 45 and 64% of women, respectively, dropped out over the pregnancy period), the 3-IPT High Strategy became the preferred strategy.

When mortality benefits were excluded from receiving CTX and ART rather than ART only, the CTX Strategy was still the most effective strategy, averting more DALYs than the 3-IPT High Strategy, and was cost-saving. In this case, the CTX Strategy averted 382 (95% CI 374–330) more DALYs than the 3-IPT High Strategy per 10,000 pregnant women.

When pregnant women started CTX in the second trimester (rather than in the first trimester as assumed in the base case for the CTX Strategy), the 3-IPT High Strategy was more effective and cost-effective than the CTX Strategy, averting 1503 (95% CI 1495–1511) more DALYs per 10,000 women at a cost of $7905 (95% CI 7866–7944), for an ICER of $5.3 (95% CI 5.2–5.3) per DALY averted.

**Fig. 3:**
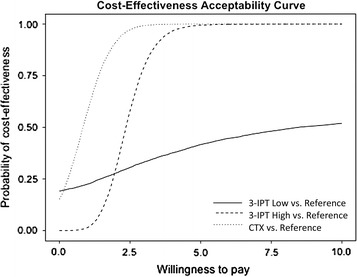
Cost-effectiveness acceptability curve for Malawi. Willingness to pay (WTP) is expressed in 2015 US dollars per DALY averted. Each line represents the probability of each strategy being cost-effective compared to the Reference Strategy (2-dose IPTp-SP) for different WTP thresholds

In probabilistic sensitivity analyses, it was found that at all willingness-to-pay (WTP) thresholds, the CTX Strategy was always preferred. Results for Malawi are shown in Fig. 3. In Malawi, for a WTP < $2.04 per DALY averted, the 3-IPT Low Strategy was more often cost-effective (compared to the Reference Strategy) than the 3-IPT High Strategy. For a WTP greater than $9.00 per DALY averted, the 3-IPT High Strategy and CTX Strategy were always cost-effective compared to the Reference Strategy.

## Discussion

Based on the analysis of the study, in regions of sub-Saharan Africa with high HIV and malaria burden, administering daily cotrimoxazole to HIV-infected pregnant women regardless of CD4 cell count is the preferred strategy under most conditions. The CTX Strategy dominates the 3-IPT High Strategy by averting more DALYs at less cost in most countries. The sensitivity analyses indicate that the CTX Strategy is the most effective strategy over a wide range of assumptions about key parameter values.

The results indicate that daily CTX for HIV-infected pregnant women would generate substantial reductions in the incidence of three primary health outcomes—maternal anaemia, malaria parasitaemia, and LBW—that cause avertable disease burden in pregnant women and infants in malaria endemic regions. Additionally, the CTX Strategy yielded ICERs ranging from cost-saving to $3.9 compared to the Reference Strategy—far below WTP thresholds in the studied countries. These results hold as long the weekly CTX dropout rate is < 0.5% (equivalent to 18% over the entire pregnancy period) in high-incidence countries (Malawi and Mozambique) and < 1.5% (45% over the entire pregnancy period) in low-incidence countries (Tanzania, Kenya, and Ghana). A recent clinical trial found that fewer than 10% of HIV-infected pregnant women reported incomplete adherence to CTX [44]. Thus, the results of the current study suggest that the CTX Strategy is likely to be the preferred strategy over a reasonable range of incomplete adherence rates [44].

The findings from the study support the updated WHO guidelines that recommend continued use of CTX regardless of CD4 cell count where malaria is common [8]. While the results are contingent upon initiation of CTX earlier in the course of pregnancy and adherence to the daily CTX dose, given currently available data on the efficacy of each prevention strategy and associated cost estimates, CTX is likely to be the preferred strategy. The cost estimates are conservative, as cost savings associated with reduced maternal anaemia, malaria parasitaemia, and LBW were not included due to lack of data on specific breakdown of the costs. If associated cost savings were included, the CTX Strategy would look even more favourable compared to the 3-IPT High Strategy.

This analysis has several limitations. First, we modeled the effects of each strategy based on published meta-analytic data and results from randomized trials [19, 21, 29, 45]. Due to lack of data directly comparing the prevention strategies, results from randomized trial and meta-analytic data were combined, and the relative risks/benefits of each strategy compared to the Reference Strategy were calculated. In addition, the meta-analytic data had some potential sources of bias, which could have led to overestimation of the effectiveness of the 3-IPT strategies [29]. Second, the rate of malaria infection was modeled as a function of CD4 cell count and preventive treatment status, and malaria parasite transmission by mosquitoes was not modeled because the interventions would not differentially affect the transmission dynamics in the homogeneously simulated population, and thus malaria transmission risks. Also, the model was simulated for a short period of time, which was unlikely to have major impact on parasite dynamics. However, inclusion of the dynamics of malaria infection is unlikely to change our results, other than potentially increasing the estimated benefits from the preventive treatment.

While the model results were quite robust to varying levels of model parameters, the lack of head-to-head comparisons of CTX and IPTp-SP suggests that a randomized controlled trial assessing the efficacy of CTX compared to 3 doses of IPTp-SP could be informative, particularly in areas with low malaria transmission, and could guide future modeling studies of the cost-effectiveness of these interventions.

## Conclusions

A strategy of providing daily CTX to HIV-infected pregnant women in malaria-endemic regions is generally more effective and less costly than strategies that provide 2 or 3 doses of IPTp-SP. Administering CTX in addition to ART for all HIV-infected pregnant women in such areas, regardless of CD4 count, would not only be cost-effective but also feasible to implement.

## Additional file

**Additional file 1.** Appendix.
