# Supplementary material for: Cost-effectiveness of malaria preventive treatment for HIV-infected pregnant women in sub-Saharan Africa
Source: Malar J. 2017 Oct 6;16:403. doi: 10.1186/s12936-017-2047-x (PMC6389090; doi:10.1186/s12936-017-2047-x)
Supplement: Supplementary file 1 — Additional file 1. Appendix. [file 12936_2017_2047_MOESM1_ESM.docx]

**Additional file 1**

**Figure S1. Model schematic**


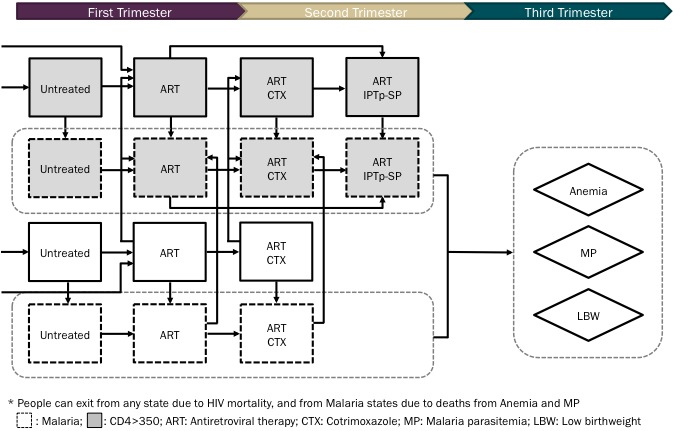


**Simulation code available at** https://github.com/sungeunc/

**Table S1. Sensitivity Analysis: Adherence to CTX**

| **Weekly Dropout Rate from CTX** | **Costs per 10,000 Women (USD)** | **DALYs per 10,000 Women** | **ICER (CTX vs. Reference Strategy)** | **ICER (3-IPT High vs. CTX)** |
| --- | --- | --- | --- | --- |
| **Ghana** |  |  |  |  |
| 0.0% | 361,009 | 9,312 | 0.37 | Dominated |
| 0.5% | 360,706 | 9,686 | 0.30 | Dominated |
| 1.0% | 360,565 | 10,060 | 0.29 | -0.75 |
| 1.5% | 359,837 | 10,459 | Cost-saving | -1.80 |
| 2.0% | 359,426 | 10,914 | Cost-saving | -4.17 |
| 2.5% | 359,128 | 11,134 | Cost-saving | -8.22 |
| 3.0% | 358,798 | 11,477 | Dominated | ̶ |
| 3.5% | 358,609 | 11,694 | Dominated | – |
| **Malawi** |  |  |  |  |
| 0.0% | 396,580 | 11,317 | 0.84 | Dominated |
| 0.5% | 396,380 | 11,978 | 0.97 | Dominated |
| 1.0% | 396,187 | 12,603 | 1.17 | 13.51 |
| 1.5% | 395,449 | 13,067 | 1.05 | 5.44 |
| 2.0% | 395,316 | 13,550 | 1.38 | 3.30 |
| 2.5% | 394,700 | 13,978 | 1.34 | 2.80 |
| 3.0% | 394,350 | 14,356 | 1.83 | 2.44 |
| 3.5% | 394,078 | 14,707 | Dominated | – |
| **Kenya** |  |  |  |  |
| 0.0% | 386,518 | 5,339 | 1.99 | Dominated |
| 0.5% | 386,294 | 5,531 | 2.10 | Dominated |
| 1.0% | 386,090 | 5,805 | 2.40 | Dominated |
| 1.5% | 385,626 | 6,068 | 2.59 | Dominated |
| 2.0% | 385,118 | 6,359 | 2.97 | 19.60 |
| 2.5% | 384,789 | 6,499 | 3.14 | 11.94 |
| 3.0% | 384,329 | 6,685 | 3.53 | 8.44 |
| 3.5% | 384,309 | 6,842 | Dominated | – |
| **Mozambique** |  |  |  |  |
| 0.0% | 404,816 | 15,240 | Cost-saving | Dominated |
| 0.5% | 404,513 | 15,906 | Cost-saving | Dominated |
| 1.0% | 404,252 | 16,573 | Dominated* | – |
| 1.5% | 403,463 | 17,291 | Dominated* | – |
| 2.0% | 403,197 | 17,796 | Dominated* | – |
| 2.5% | 402,885 | 18,327 | Dominated* | – |
| 3.0% | 402,242 | 18,854 | Dominated* | – |
| 3.5% | 402,241 | 19,225 | Dominated* | – |
| **Tanzania** |  |  |  |  |
| 0.00% | 371,154 | 3,516 | 3.85 | Dominated |
| 0.5% | 370,794 | 3,641 | 4.02 | Dominated |
| 1.0% | 370,331 | 3,821 | 4.50 | Dominated |
| 1.5% | 369,844 | 3,966 | 4.90 | Dominated |
| 2.0% | 369,256 | 4,147 | 6.27 | Dominated |
| 2.5% | 368,981 | 4,232 | 8.25 | 2,487 |
| 3.0% | 368,643 | 4,390 | Dominated | – |
| 3.5% | 368,385 | 4,531 | Dominated | – |

CTX = cotrimoxazole; DALY = disability-adjusted life year; ICER: Incremental cost-effectiveness ratio (cost per DALY averted); IPT = intermittent preventive treatment with sulfadoxine pyrimethamine

*Saves costs compared to the Reference Strategy but incurs more DALYs

**Figure S2. Sensitivity analysis on adherence rate to CTX (percent of women who drop out each week)**

*Reference Strategy
